# Health economic evaluation of an interdisciplinary care pathway for older patients with vertigo, dizziness and balance disorders in primary care (MobilE-PHY2) - a cluster-randomised trial

**DOI:** 10.1186/s12962-026-00779-0

**Published:** 2026-06-04

**Authors:** Viola Zimmer, Caren Horstmannshoff, Petra Bauer, Peggy Borchers, Sandy Scheibe, Karen Voigt, Martin Müller, Juliane Köberlein-Neu

**Affiliations:** 1 Centre for Health Economics and Health Service Research, University of Wuppertal, Rainer- Gruenter-Str. 21, 42119 Wuppertal, Germany; 2 TUM School of Medicine and Health, Technical University of Munich, Georg- Brauchle-Ring 60/62, 80992 Munich, Germany; 3 Centre for Research, Development and Technology Transfer, Rosenheim Technical University of Applied Sciences, Hochschulstr. 1, 83024 Rosenheim, Germany; 4 Faculty of Applied Health and Social Sciences, Rosenheim Technical University of Applied Sciences, Hochschulstr. 1, 83024 Rosenheim, Germany; 5 Department of General Practice, Faculty of Medicine, University Hospital Carl Gustav Carus, TUD Dresden University of Technology, Fetscherstraße 74, 01307 Dresden, Germany; 6 Nursing Science and Interprofessional Care, Department for Primary Care and Health Services Research, Medical Faculty Heidelberg, Heidelberg University, Heidelberg, Germany

**Keywords:** Dizziness, Vertigo, Balance disorder, Care pathway, Health economic evaluation, Primary care, Physiotherapy

## Abstract

**Background:**

Vertigo, dizziness and balance disorders affect over half of adults aged 65 and older. The MobilE-PHY2 study, conducted in Germany, evaluated the effectiveness of an evidence-based, multidisciplinary care pathway for this population. From a health economic perspective, the intervention was expected to increase outpatient and physiotherapy costs while reducing resource utilisation in other healthcare areas and improving patient-relevant outcomes.

**Objective:**

This health economic evaluation examined the cost-effectiveness of the care pathway compared to optimised routine care. Incremental costs were related to incremental changes in both quality-adjusted life years (QALYs) and the Dizziness Handicap Inventory (DHI) as a disease-specific outcome; expressed as incremental cost-effectiveness ratios (ICER).

**Methods:**

The study was a multicentre, cluster-randomised controlled trial with six -month follow-up. The health economic evaluation was conducted from a societal perspective. Cost-effectiveness was determined via incremental cost-utility ratios (ICURs) and incremental cost-effectiveness ratios (ICERs). Uncertainty was addressed using cost-effectiveness acceptability and net-monetary-benefit curves. Missing data were handled using Multiple Imputation by Chained Equations. Sensitivity analyses excluded informal care costs and focused on a subgroup with reduced imputation uncertainty.

**Results:**

The ICER was 81,246.85€ per additional patient achieving a clinically relevant DHI improvement. Indicating that if the willingness-to-pay threshold were €50,000 per additional patient achieving a clinically relevant DHI improvement, the intervention would be cost-effective with 71% probability. The ICUR was negative (-121,847.54€/QALY), meaning optimised routine care dominated the intervention. From a payer perspective, the intervention was less costly, though with lower QALYs. Findings were robust in sensitivity analyses.

**Conclusion:**

The care pathway shows potential for cost-effectiveness among patients achieving DHI improvements, supporting cautious, targeted implementation in routine care. Future research should address the economic burden on informal caregivers.

**Trial registration:**

DRKS00028524 retrospectively registered on March 24, 2022.

**Supplementary Information:**

The online version contains supplementary material available at 10.1186/s12962-026-00779-0.

## Introduction

Dizziness and vertigo are common complaints in clinical practice, with a reported prevalence of up to 50% that increases with age [1–3]. Dizziness broadly refers to a range of atypical perceptual experiences concerning the body’s orientation in space [4]. In contrast, vertigo specifically describes a false sensation of movement, typically a spinning sensation, either of the environment or the body, or light headedness [5]. The aetiology of dizziness and vertigo is diverse, ranging from cardiovascular and peripheral otological causes, such as benign paroxysmal positional vertigo and vestibular neuritis, to cases where no specific diagnosis can be established [5]. Frequently, the underlying cause is multifactorial, further complicating diagnosis and management [6].

The physical and psychological impacts associated with dizziness and vertigo include immobility, limitations in activities of daily living, decreased social participation, and lower psychological well-being [7]. Vertigo is recognised as a distinct risk factor for falls [8]. Among individuals with vestibular dysfunction, 69.8% reduced their workload, and 5.7% report resigning from their jobs due to vertigo-related symptoms [9]. Furthermore, the economic burden on patients and healthcare providers is considerable [10], driven by repeated consultations and the excessive utilisation of diagnostic imaging [11]. In particular, the management of vertigo and dizziness in primary care settings is associated with considerable healthcare costs, highlighting this sector as a strategically relevant point of intervention [12]. This is underlined by research showing that dizziness is one of the most frequent reasons for consulting a general practitioner (GP), with prevalence estimates of GP consultations due to dizziness reaching up to 15.5% [5, 13]. Despite its high prevalence, establishing a definitive diagnosis remains a significant challenge in the management of dizziness and vertigo, emphasising the need for effective, evidence-based diagnostic tools [14].

A multidisciplinary care pathway (CPW) was developed and piloted to address this need, integrating evidence-based approaches for the management of vertigo, dizziness and balance disorders (VDB). Despite challenging recruitment, the pilot study demonstrated feasibility and acceptability of the intervention as well as potential benefits for patients. Patients responded well to physiotherapy, with good treatment adherence reported [15, 16]. These findings suggest that a structured, multidisciplinary approach could enhance the management of VDB in primary care settings. In this health economic evaluation alongside a cluster-randomised clinical trial (cRCT) [17], we investigate if the evidence-based CPW is cost-effective in terms of the incremental costs per:

1. Incremental patient who experienced a clinically meaningful decrease of at least 12 points in the Dizziness Handicap Inventory (DHI) questionnaire during the study period [18], and
2. Incremental quality-adjusted life years (QALYs) gained during the study period.

## Methods

This health economic evaluation is based on the Consolidated Health Economic Evaluation Reporting Standards (CHEERS) [19] in conjunction with the ISPOR-CEA recommendations for conducting cost-effectiveness analysis in the context of clinical trials [20]. The focal point of this publication is the cost-effectiveness and cost-utility analysis of the CPW.

### Study design

The MobilE-PHY2 study was a pragmatic, controlled, multicentre, cluster-randomised, cluster-blinded trial with two parallel groups and a 1:1 allocation ratio. The study was conducted in two regions, each characterised by an urban centre with a surrounding rural environment. The objective of the trial was to evaluate the effectiveness and safety of the evidence-based multidisciplinary CPW improving mobility and participation in community-dwelling older patients with VDB in primary care [17]. The CPW intervention was compared to a control group receiving optimised routine care. Based on findings from the pilot study, which included interviews with patients, modifications were made to the study design and implementation processes to enhance recruitment, streamline procedures, and optimise intervention delivery.

### Sample and recruitment

Community-dwelling patients aged 60 years or older who had presented in the previous three years to their GP with either recent or chronic complaints of VDB were deemed eligible to participate. Patients were required to be able to stand up on their own and maintain a standing position for two minutes with support. They were excluded from participation if they had not given written informed consent, had a DHI score of less than 12 points, exhibited moderate to severe cognitive impairment (Mini-Mental State Examination (MMSE) score of less than 20), had a psychiatric disorder based on specific ICD codes (F10.-F19, F20.-F29, F30., F31., F32.2, F32.3, F32.8, F32.9, F33.2, F33.3, F33.8, F33.9), had a limited life expectancy of less than one year, had a current substance abuse as a cause of VDB, or had insufficient command of the German language [17].

The recruitment of all participants was conducted between 09/2021 und 10/2022. Patients eligible to participate were provided with written and verbal study information regarding their involvement prior to providing written consent. Participating GPs and physiotherapists (PTs) were required to hold accreditation with German statutory health insurances. Additionally, GP practices were required to have practice software capable of identifying potential study participants. Participating GP practices received a case payment of €60 per patient, while PT practices received €30 per patient. GPs were randomly allocated to either the intervention or control group using block randomisation, stratified by region, with GPs serving as the cluster level for the randomisation process and further analyses [17].

### Intervention and control

A logic model was developed for the intervention [17], outlining key components for both professionals and patients. The intervention aimed to equip GPs and PTs with the necessary knowledge and skills to support patients in improving their mobility and participation. By improving patient self-management, the intervention was expected to reduce the need for healthcare resources, ultimately leading to lower healthcare costs.

The intervention consisted of an evidence-based, algorithmized CPW designed to improve mobility and participation in older patients with VDB. To summarize the CPW, patients presenting to their GP with VDB symptoms were diagnosed and treated using a checklist and, if deemed necessary, referred to a specialist, or a PT who applied a decision tree. Patient-facing components included a structured assessment, tailored advice, and targeted physiotherapy aimed at improving balance, mobility, and daily functioning. Patients were actively engaged in their care through guidance on self-management strategies. Follow-up consultations with the GP were scheduled at 4 weeks and 3 months to monitor progress and adjust care as needed (see Appendix 1 for full details).

To implement the intervention, GPs participated in a two-hour online educational training, while PTs attended a one-day training session. The paper-based checklist to standardise GPs clinical decision-making processes encompassed diagnostics, treatment, and referral options throughout the treatment period. The decision tree provided to PTs guided their evidence-based clinical reasoning, assessment, treatment, and evaluation of patients [17].

The control group was offered 30 mins of online training on the German national guideline on acute dizziness to mask the GPs’ allocation to the groups, participation in the online training was voluntary [17]. Refer to Appendix 2 for a detailed description of the implementation strategies.

### Data collection

The information relevant to the health economic evaluation was collected at the patient level before the intervention ($\:{t}_{0}$, baseline) and 6 months after the start of the intervention ($\:{t}_{2}$, follow-up). Participants completed paper-and-pencil questionnaires either autonomously or with the assistance of their caregivers. Data were pseudonymised and protected in accordance with the EU General Data Protection Regulation and federal legislation.

### Measures and outcomes

#### Primary outcome

The Dizziness Handicap Inventory - German Version is a condition-specific instrument designed to evaluate self-perceived disability in older patients with particular regard to limitations in mobility, activity and participation [21]. It was used as the basis for calculating the outcome used in the cost-effectiveness analysis of this health economic evaluation [17]. Patients rated their degree of disability using 25 items with a scale ranging from “no” (0 points) and “sometimes” (2 points”) to “yes” (4 points), for a total of 100 points, with 100 indicating the highest level of self-perceived handicap. For the health economic evaluation, this score was reversed so that zero indicates the most severe disability, enabling interpretation akin to QALYs (i.e. more is better). The German version has been shown to exhibit both high reproducibility and good internal consistency [21, 22]. A mean reduction of 12 points between baseline ($\:{t}_{0}$) and follow-up ($\:{t}_{2}$) is considered a clinically relevant improvement [18] and will be referred to hereafter as “DHI improvement”. Participants meeting this threshold were classified as having a positive outcome, while those who did not were categorized as not achieving the outcome. The binary indicator was used as the effectiveness outcome in our analysis. As there is no established WTP threshold for DHI improvements, a threshold of €50,000 per DHI improvement was applied, in line with upper ranges cited for the United Kingdom [23], as Germany does not apply a fixed threshold. While this transfer is not formally validated, it provides a pragmatic reference point to aid interpretation and comparability across outcome measures.

#### EQ-5D-5 L and QALY

The European Quality of Life Five-Dimension Five-Level scale (EQ-5D-5L) was considered in this health economic evaluation as the basis for measuring utility [24]. The results of this scale were evaluated using German tariffs to obtain EQ-5D index values using the German Value Set for the EQ-5D-5L contained in the R package eq5d [25, 26]. QALYs were calculated using the area under the curve method, applying linear interpolation between baseline and follow-up EQ-5D index values, weighted by the duration of the interval.

#### FIMA

The Questionnaire for Health-Related Resource Use in an Elderly Population (German: *Fragebogen zur Inanspruchnahme medizinischer und nicht-medizinischer Versorgungsleistungen im Alter*, FIMA) is a tool designed to assess health-related resource use in older populations [27]. The questionnaire consists of 26 items and utilises varying recall periods, depending on the salience of events. For instance, contacts to physicians are less distinctive and necessitate shorter recall periods, whereas hospital visits tend to be recalled well even after a longer period. The validity of the measurement of PT visits, in particular, has been substantiated by FIMA [27].

#### Unit cost

Resource usage was valued using standardised unit costs in accordance with Bock et al. [28], with a recent update provided by Muntendorf et al. [29]. These publications provide nationally representative unit cost estimates based on routinely available data from insurance providers to ensure comparability across economic evaluations. Medical devices and dentures were categorized into their product categories, with each category being counted once per patient if at least one item was reported, regardless of the number of items received. Informal care was measured using self-reports and valued using a replacement cost approach. Medication costs had to be excluded from both perspectives due to a lack of consistent information on patient’s medication. Table 1 provides detailed information on the monetary valuation of resources, as well as their units and recall periods used by FIMA. Information on the costs of implementation, especially GP and PT training was not available. All costs were calculated in 2023 euros, and values from questionnaires completed in 2022 were adjusted for inflation using the German consumer price index [30]. Costs were not discounted due to the short time frame of six months. Productivity losses were not included in total costs, as most of the population is of retirement age (mean age: 78 years).

First, we adopt a societal perspective combining societal resource use, while excluding medication costs due to data limitations. In addition, we assume a payer perspective, excluding informal care costs and care allowances. This approach provides a comprehensive view of the economic consequences of the intervention for patients, caregivers, and payers within the constraints of available data.

**Table 1:** Cost categories and unit costs, all unit costs sourced from Muntendorf et al. [29]

Cost category	Service	Recall period [months]	Unit Costs € in 2023	Unit
Outpatient physician services	General practitioner	3	29.98	Contact
	Gynaecologist	3	48.27	Contact
	Internal medicine specialist	3	85.35	Contact
	Surgeon	3	61.52	Contact
	Orthopaedics	3	37.10	Contact
	Neurology	3	70.80	Contact
	Dermatology	3	29.96	Contact
	Ophthalmology	3	60.61	Contact
	Urology	3	34.71	Contact
	Dentistry	3	61.68	Contact
	Outpatient hospital care	3	767.55	Day
	Physiotherapy	3	25.03	Contact
Outpatient non-physician services	Occupational therapy	3	63.27	Contact
	Speech therapy	1	70.85	Contact
	Medical foot care	3	44.33	Contact
Ambulatory care and support (professional)	Nursing services	3	0.69	Minute
	Domestic assistance	3	0.49	Minute
Nursing home care	Inpatient short-term care Long-term care grade 1	3	74.09	Day
	Inpatient short-term care Long-term care grade 2–5		188.9	
	Partial inpatient nursing care Long-term care grade 1	3	39.44	Day
	Partial inpatient nursing care Long-term care grade 2		54.96	
	Partial inpatient nursing care Long-term care grade 3		78.02	
	Partial inpatient nursing care Long-term care grade 4		90.37	
	Partial inpatient nursing care Long-term care grade 5		105.07	
Informal care	Informal care	3	35.32	Hour
Rehabilitative care	Outpatient rehabilitative care	12	87.48	Day
	Inpatient rehabilitative care	12	215.70	Day
Inpatient services	General hospitals: normal ward	12	1,180.84	Day
	General hospitals: intensive care unit		2,559.00	Day
Medical devices and dentures	Walking aid	12	70.71	Category reported at least once
	Wheelchairs		1,058.22	
	Bath aids (e.g. bathtub lift)		122.24	
	Visual aid (e.g. glasses)		101.87	
	Hearing aid		1,542.40	
	Inhalation and respiratory therapy devices (e.g. oxygen device)		664.94	
	Aid for compression therapy		273.25	
	Incontinence pads		376.32	
	Dentures		736.18	Case

#### Additional variables

Other covariates used in regression analyses were the Barthel-Index (BI) and the Mini-Balance Evaluation Systems Test (MiniBESTest) score. The German version of the MiniBESTest was used to assess static and dynamic balance using 14 items, with each item being scored from 0 to 2, and higher scores indicating better postural control. The MiniBESTest demonstrates a good validity and internal consistency [31].

The BI has been used to assess the independence in basic daily activities and has good reliability in non-cognitively impaired populations [32, 33]. Patients rated themselves on 10 items with different response scales and a maximum total score of 20, with higher scores indicating greater dependence.

### Statistical analysis

Primary analyses were based on the intention-to-treat (ITT) principle. Cost-effectiveness and cost-utility analyses were performed from both a societal perspective and a payer perspective for the 6-month cRCT applying linear mixed models. A 5% significance level was used for all statistical analyses, unless otherwise stated. All analyses were performed with R version 4.3.3 [34]. Due to the overall small sample size and the relatively small cluster sizes, all analyses were adjusted for sex, age, standardised baseline costs, BI and MiniBESTest score. Adjusting for comorbidities not reflected by these scores would have been desirable, however, no data on patients’ underlying conditions were collected. Baseline costs, reflecting healthcare expenditures incurred in the 12 months preceding the intervention, exhibited substantial skewness and large scale; they were therefore z-score standardised by mean-centring and scaling to unit variance. All linear mixed models included the primary care practice as a random effect. There was no pre-specified statistical analysis plan, and patients were not involved in designing the health economic evaluation.

#### Imputation of missing values

Missing data were imputed separately for the intervention and control groups using Multiple Imputations by Chained Equations (MICE) [35]. Cost data were imputed at the item level for each time point, as were EQ-5D utilities, DHI, BI, and MiniBESTest scores. There were 16 participants without any measurement at t_2_ for whom resource use and outcome measures had to be imputed within the MICE framework using available baseline data. The mean percentage of missing data per variable was 12.3%, with 5% of variables having more than 20% missing data (standard deviation = 7.5%, minimum = 0%, maximum = 51.5%). Following the recommendations of Graham et al. [36], 20 data sets were generated after five iterations each. Convergence was assessed at both five and forty iterations, with satisfactory convergence reached at five iterations. Data were assumed to be missing at random (MAR). Continuous variables were imputed using predictive mean matching. Due to the presence of collinearity, days spent in outpatient care, psychiatric or rehabilitation clinics and short-term care, as well as supplemental oxygen devices, were excluded from the analysis. This resulted in a predictor matrix containing all variables included in subsequent analyses. Data for the intervention and control groups were imputed separately [37]. After imputation, analyses were performed for each imputed dataset, and the results were pooled according to Rubin’s rules [38].

#### Calculation of costs and QALYs

The time under intervention was defined as the number of days between completing the study’s questionnaires at baseline (t_1_) and follow-up (t_2_). Unadjusted total costs at follow-up ($\:{C}_{i}$) were calculated by multiplying individual total costs at follow-up by the proportion of the year spent under intervention. To account for baseline cost differences, costs from the 12 months before the intervention were estimated using the FIMA questionnaire at $\:{t}_{0}$.

QALYs were adjusted based on the duration of the intervention using linear interpolation (area-under-the-curve method) between baseline and follow up EQ-5D valuation indices. Further information on the calculation of costs and QALYs is available from Appendix 3.

#### Comparison of baseline characteristics

Baseline characteristics of the intervention group (IG) and control group (CG) are compared using descriptive statistics for both original and imputed data, univariate mixed effects models were used to identify differences at baseline in the imputed data. To accommodate the requirements of the gamma distribution, which does not allow for values of zero, the single patient with zero costs was recoded to a value of €0.01.

#### Cost-effectiveness and cost-utility analysis

In a first step we calculated descriptively the mean costs and the mean effects (based on the DHI Score of the cRCT and on the EQ-5D-5L index) per group. The difference in costs between IG and CG were adjusted using generalised linear mixed models (GLMM) with a gamma distribution and a log-link function. For utility differences, we used QALYs as the dependent variable, while effectiveness was assessed using the binary indicator for a DHI improvement described above. Both GLMMs assumed a Gaussian distribution with an identity link function, incorporating the EQ-5D-5L valuation index (utility) and the baseline DHI Score (effectiveness) as additional independent variables.

To provide point estimates for cost-utility, the incremental cost-utility ratio (ICUR) was computed as the ratio of the estimated mean costs to the estimated mean difference in QALYs for the intervention group, providing a standardised measure of cost per QALY gained. Similarly, the incremental cost-effectiveness ratio (ICER) was derived using the mean difference in proportion of people with a DHI improvement in the intervention group as the denominator, representing cost per DHI improvement. As data was imputed, the coefficients were calculated separately for each data set and then pooled using Rubin’s Rules [38]. Confidence intervals for the ICER and the ICUR were estimated using a bootstrap procedure applied to the covariate-adjusted predictions from GLMMs. Specifically, for each of the 20 imputed datasets, 1,000 bootstrap samples were drawn with replacement, preserving the original sample size. Separate GLMMs were then fitted on each bootstrap sample for costs and QALYs, including covariates and clusters from the main models. Adjusted mean predictions for each treatment group were derived from the fitted models using marginal effects, and the incremental cost and incremental QALY differences were computed as the contrast between groups. The bootstrap was thus applied to model-based predictions rather than raw observed values, and uncertainty estimates retain the parametric distribution assumptions of the GLMMs. The ICER was then calculated for each of the resulting 20,000 bootstrap replications. Bias-corrected and accelerated (BCa) confidence intervals were calculated separately within each imputed dataset and subsequently pooled across imputations following the MI-Boot approach [39]. The results are visualised in cost-effectiveness planes (CE-planes), which are divided into four quadrants based on cardinal directions: The north-east quadrant represents scenarios where both cost and effect differences are positive, while the south-east quadrant indicates favourable effects at lower cost (positive x, negative y). The south-west quadrant reflects reduced costs but also unfavourable effects (negative x and y), and the north-west quadrant captures cases where costs increase but effects are higher (negative x, positive y).

#### Decision uncertainty

To evaluate the uncertainty of the cost-effectiveness estimates, cost-effectiveness acceptability curves (CEACs) were calculated. A CEAC reflects the probability that the intervention is more cost-effective than optimised routine care, if the decision-maker is willing to pay a certain threshold value ($\:{R}_{T}$) for an additional QALY or an additional patient with a DHI improvement. The CEAC is derived by multiplying different values of $\:{R}_{T}$ with effect differences and comparing these to cost differences. Each value of $\:{R}_{T}$ effectively separates the CE-plane into two surfaces: one above and another below the price line [40]. Above this price line, the intervention is not cost-effective; conversely, the proportion of bootstrapped ICURs and ICERs below the price line indicates the likelihood that the intervention’s cost is acceptable to the decision maker.

#### Sampling uncertainty

Sampling uncertainty was quantified using Net Monetary Benefit (NMB) regressions, with the calculation of individual net monetary benefit being conducted for willingness-to-pay (WTP) threshold values ($\:{R}_{T}$) ranging from 0 up to 150,000 in steps of 1,000. The individual net monetary benefit was calculated using the following formula:

$$\:NM{B}_{i}=\delta\:{E}_{i}\cdot\:{R}_{T}-\delta\:{C}_{i}$$

$\:NM{B}_{i}$ was the dependent variable in a GLMM, with group as the independent variable, and age, gender, BI and MiniBESTest score as further covariates. Clustering was addressed through the incorporation of random effects for primary care practices. The GLMM employed a Gaussian distribution assumption and the coefficient of group is reported for each $\:{R}_{T}$ utilising NMB curves.

#### Sensitivity analysis

We conducted a sensitivity analysis for cases with completed questionnaires at both baseline and follow-up. For this population with two questionnaires (TQ), the ITT was narrowed to include only participants who had completed the $\:{t}_{2}\:$questionnaire, resulting in the exclusion of 16 cases that did not have $\:{t}_{2}\:$data. Imputation was then repeated for the remaining 82 participants, and the updated data were used for all subsequent analyses. The main purpose of the TQ subgroup is verifying MICE, which had to handle 12.3% missing data in the ITT population, compared to 3.6% for the TQ.

## Results

### Characteristics of the study population at baseline

The mean age of participants was 78 years (SD: 8.18, range: 60–94) and about three quarters were female (71%). The mean BI was 18.28 (SD: 2.43, range: 8–20) and the mean MiniBESTest Score was 16.48 (SD: 6.65, range: 0–28). Standardised baseline costs were observed to be higher in the IG (mean: 0.89, SD: 5.40) than in the CG (mean: -1.13, SD: 1,43), a discrepancy that can be attributed to the difference in informal care cost, which was one of two statistically significant estimators, the second being medical aids and dentures (Table 2).

**Table 2:** Comparison of effect, costs and covariates at baseline; CG: control group, DHI: Dizziness Handicap Inventory, EQ5D: European Quality of Life Five-Dimension Five-Level scale, IG: intervention group, MiniBESTest: Mini-Balance Evaluation Systems Test, sd: standard deviation

	Total Sample	IG	CG
**Covariates**			
Number of participants	98	55	43
Gender (% female)	71	74	67
Age, years (mean(sd))	78.45 (8.18)	78.35 (7.89)	78.54 (8.54)
Standardised baseline cost (mean(sd))	0.00 (4.26)	0.89 (5.40)	-1.13 (1.43)
Barthel Index (mean(sd))	18.28 (2.43)	18.00 (2.55)	18.62 (2.26)
MiniBESTest Score (mean(sd))	16.48 (6.65)	15.47 (6.84)	17.84 (6.22)
Equation 5D valuation index at t_0_ (mean(sd))	0.0 (0.28)	0.68 (0.30)	0.73 (0.25)
DHI Score (mean(sd))	52.57 (22.57)	47.78 (23.79)	58.70 (19.52)
**Costs [€]** mean(sd)			
Outpatient care (without primary care)	1,751.97 (2,266.40)	2,057.16 (2,695.66)	1,361.62 (14,97.42)
Primary care	329.33 (367.34)	341.95 (258.24)	312.80 (477.11)
Rehabilitation	431.31 (1,433.15)	592.54 (1,739.53)	225.08 (880.60)
Physiotherapy	456.59 (555.85)	611.78 (604.98)	261.70 (417.75)
Outpatient non-physician services (without physiotherapy)	307.63 (1,123.56)	405.53 (1,394.14)	182.41 (622.84)
Medical devices and dentures	94.59 (302.48)	114.63 (322.75)	68.95 (276.02)
Inpatient services	4,118.36 (10,402.81)	5,147.18 (11,531.00)	2,802.41 (8,707.48)
Ambulatory care and support (professional)	2,314.58 (4,336.18)	2,542.26 (4,109.39)	2,033.95 (4,634.17)
Informal care	12,401.08 (43,410.40)	20,783.48 (57,189.92)	2,069.30 (3,885.77)
**Total cost**	21,935.80 (47,450.36)	31,806.13 (60,163.49)	9,310.96 (15,980.81)

### Costs and effects

Table 3 presents the comparison of costs and effects between the IG and CG for both the ITT and TQ populations. The mean total cost difference in the ITT was €5,663.47 (CI: -1,842.36;13,169.31), compared to a mean of €5,988.12 in the TQ (95% CI; -692.76;12,669.00). Estimators for the cost difference for medical aids and remedies were elevated, but statistically not significant, likely due to the high proportion of zeroes observed in this category (ITT: 71%, TQ: 70%). Informal care cost was higher in the IG for both populations, with adjusted mean differences of €15,305.78 95% CI: (-6,566.73;37,177.94) for the ITT and €17,949.06 (95% CI: 1,821.90;34,076.22) for the TQ.

In terms of health outcomes, the ITT analysis showed that participants in the IG had, on average, 0.04 QALYs (95% CI: -0.09;0.00) less than the CG and demonstrated a lesser improvement in DHI scores, with an average increase of 1.01 points compared to the CG (95% CI: -7.60;9.61), and 6 percentage points more participants achieving a clinically relevant improvement in their DHI scores (95% CI: -0.13;0.25).

The TQ analysis revealed a more pronounced improvement in DHI scores for the IG, with an increase of 1.46 points (95% CI: -5.44;8.36) and a larger proportion of participants showing a DHI improvement (10% points, 95% CI: -0.07;0.28). In contrast to the ITT findings, the TQ analysis showed no difference in average QALY changes between the IG and CG (mean: 0.00, 95% CI: -0.04;0.05).

**Table 3:** Comparison of costs and effects for ITT and TQ populations; CI: Confidence Interval, DHI: Dizziness Handicap Inventory, ITT: Intention-to-Treat population, QALY: Quality-Adjusted Life Years

	ITT Imputed (*n* = 98)		TQ Imputed (*n* = 82)	
	Estimate	95% CI	Estimate	95% CI
**Variables1**				
Intervention cost (standardised)	0.63	(-0.08;1.35)	0.62	(-0.01;1.25)
QALY	-0.04	(-0.09;0.00)	0.00	(-0.04;0.05)
DHI change	1.01	(-8.03;11.23)	1.46	(-5.44;8.36)
% with clinically relevant DHI improvement	0.06	(-0.13;0.25)	0.10	(-0.07;0.28)
**Costs**				
Outpatient care (without primary care)	1,773.51	(-6,265.15;9,812.16)	1,240.33	(-7,007.39;9,488.04)
Primary care	4,246.25	(-2,068.98;10,561.47)	2860.98	(-2,608.85;8,330.82)
Rehabilitation	-15,984.08	(-67,357.49;35,389.34)	-14586.73	(-53,313.96;24,140.49)
Physiotherapy	7,985.26	(-3,068.19;19,038.71)	8,798.81	(-1,582.11;19,179.74)
Outpatient non-physician services (without physiotherapy)	2,781.53	(-11,297.84;16,860.90)	-399.63	(-15,755.56;14,956.31)
Medical aids and dentures	17,231.86	(-32,337.31;66,801.02)	51,397.75	(15,470.17;87,325.33)
Inpatient services	2,129.72	(-17,186.76;21,446.20)	1719.09	(-17,720.33;21,158.51)
Ambulatory care and support (professional)	-2,905.99	(-20,490.31;14,678.33)	-4,139.03	(-21,915.56;13,637.51)
Informal care	15,305.78	(-6,566.37;37,177.94)	17,949.06	(1,821.90;34,076.22)
Total cost	5,663.47	(-1,842.36;13,169.31)	5,988.12	(-692.76;12,669.00)

^1^: Variables from covariate-adjusted GLMMs, with estimates representing mean difference of intervention group to control group

### TQ population: costs and effects

For the 82 participants who completed both baseline and follow-up questionnaires, imputed total cost differences were higher in the IG compared to the CG (mean difference: €5,988.12, 95% CI: -692.76;12,669.00), as Table 3 shows. The mean total cost in the IG was €25,525.59 (SD: €6,461.27), compared to €12,016.11 (SD: €1,769.19) in the CG. Consistent with the ITT findings, informal care cost was the primary driver of this difference.

In terms of effect differences, neither QALY nor DHI outcomes reached statistical significance. However, the DHI change differed between groups: the CG showed a larger reduction in DHI scores (mean: -1.53, SD: 1.90) than the IG (mean: 0.09, SD: 1.94). Additionally, the IG reported a higher proportion of participants achieving a DHI improvement (23%) compared to the CG (15%).

### Point estimates of cost-effectiveness

**Fig. 1:**
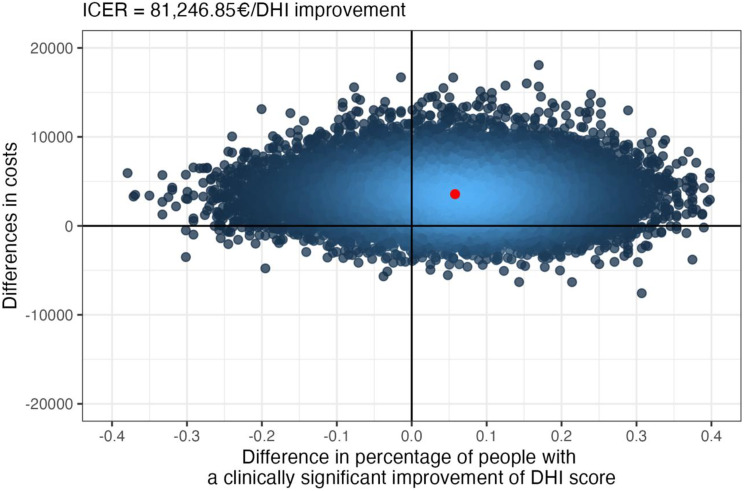
Cost-utility plane for the ITT; DHI: Dizziness Handicap Inventory

When evaluating cost-effectiveness in terms of DHI improvements, the ICER, with a value of €81,246.85 per DHI improvement, represents higher costs in the intervention group, and an increase in effectiveness. The point estimate and 71% of bootstrap replications fell in the northeast quadrant of the CE-plane (see Fig. 1).

**Fig. 2:**
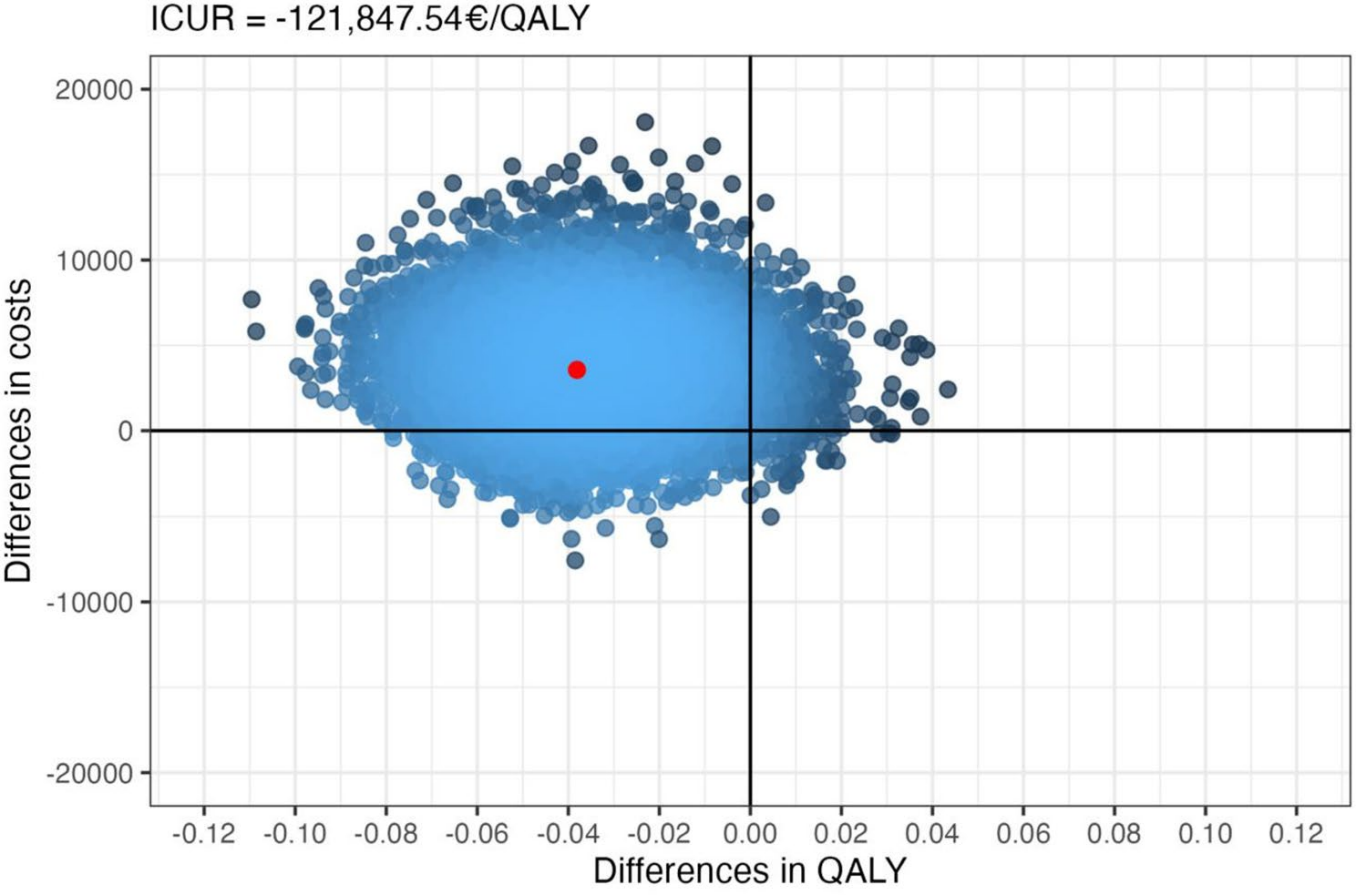
Cost-effectiveness plane for the ITT; QALY: quality-adjusted life years

In the ITT analysis with a societal perspective, the ICUR (-121,847.54 €/QALY) indicates that the intervention resulted in both higher costs and fewer QALYs compared to the CG, as the cost-effectiveness plane in Fig. 2 shows.

Excluding informal care cost and therefore assuming a payer perspective leads to a negative cost difference, showing that the intervention is less costly than optimised routine care. However, because QALYs were slightly lower compared to optimised routine care, the resulting ICUR (17,357.25€/QALY) reflects a trade-off, as cost-savings are achieved at the expense of a modest reduction in health-related quality of life. When these cost differences are considered alongside effectiveness, the ICER amounts to -11,363.76€/DHI improvement, as the intervention group showed a higher proportion of patients with improved DHI scores.

Excluding participants who completed only a single questionnaire did not meaningfully affect the results. The ICUR continued to disfavour the intervention (-222,896.27€/QALY) while the ICER was in the northeast quadrant of the cost-effectiveness plane, amounting to 55,240.31€/DHI improvement, indicating higher costs alongside improved effectiveness. Table 4 gives an overview of point estimates and bootstrap replications (*n* = 20,000) across all analyses.

**Table 4:** Cost-effectiveness planes; TQ: population with two questionnaires, CI: Confidence Interval; ICER: incremental cost-effectiveness ratio, ICUR: incremental cost-utility ratio, ITT: intention-to-treat population

Population		Point estimate	95% CI	incremental cost	incremental effects	probability cost-effective^1^
ITT (societal perspective)	ICUR	-121,848	-1,223,941;73,462	4,751.14	-0.0390	22.35%
	ICER	81,247	-1,949,824;958,460	4,751.14	0.0585	68.56%
ITT (payer perspective)	ICUR	17,357	-169,040;603,538	-665.53	-0.0383	12.98%
	ICER	-11,364	-374,147;819,867	-665.53	0.0585	69.43%
TQ	ICUR	-222,896	-4,814,395;371,834	4545.66	-0.0203	31.88%
	ICER	55,240	-3,351,637;119,326	4545.66	0.0821	78.67%

^1^: at 50,000€/QALY or DHI improvement

### Decision uncertainty

**Fig. 3:**
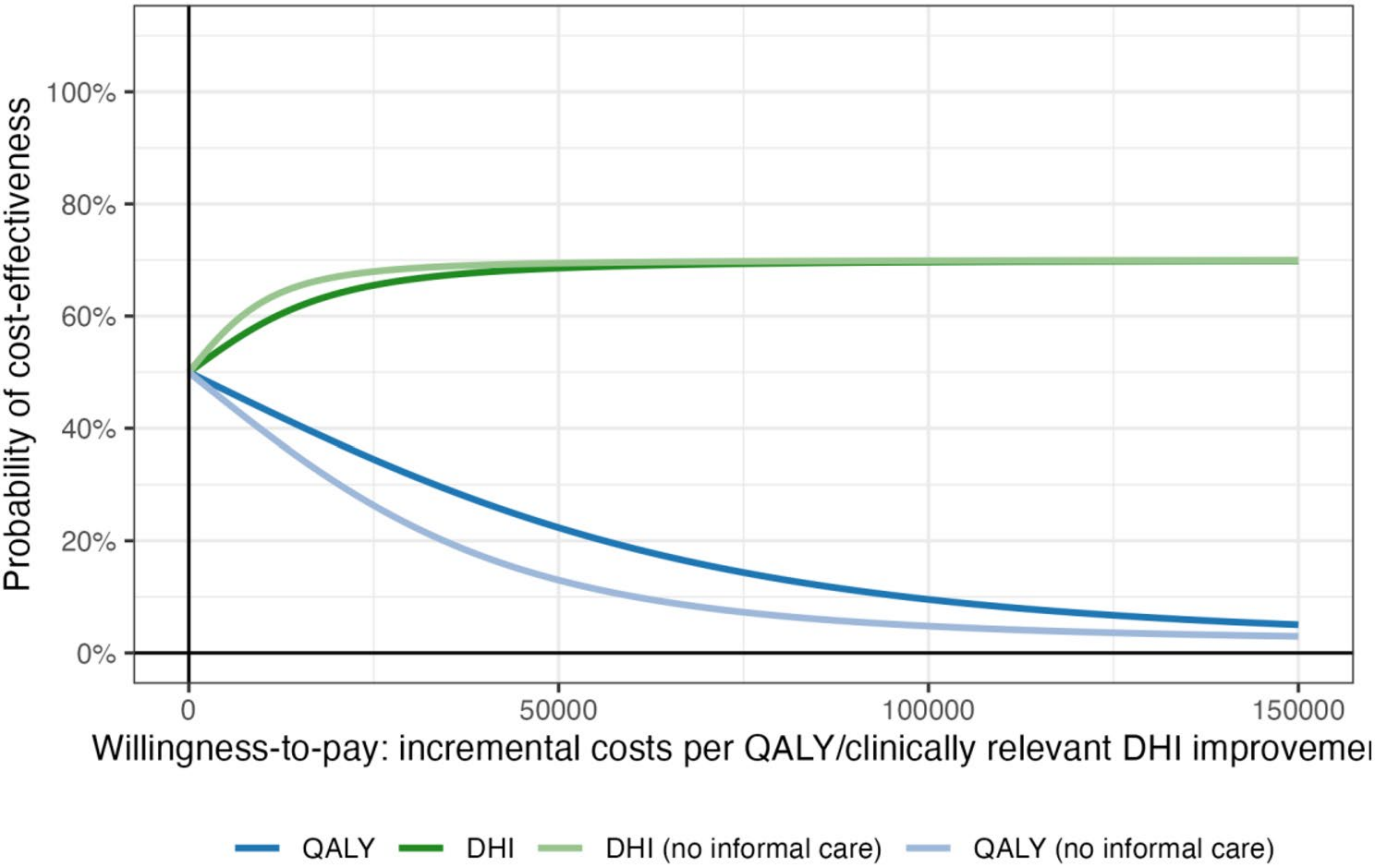
Cost-effectiveness curve for the ITT; DHI: Dizziness Handicap Inventory, QALY: quality-adjusted life years

Figure 3 presents the CEAC for the ITT analyses, both including and excluding informal care costs, for utility and disease-specific effectiveness outcomes. Due to an ICUR that reflects the higher costs and lower utility of the intervention compared to routine care, the CEAC for QALYs shows a decrease as the WTP threshold increases, meaning that at higher thresholds, the opportunity cost of the forgone QALYs is weighted more heavily, further reducing the probability that the intervention would be considered cost-effective. In contrast, the disease-specific outcome suggests potential benefits of the intervention, with both curves approaching an asymptote at 75%. At a WTP threshold of €50,000/DHI improvement, the probability that the intervention is cost-effective is 72%, compared with 74% when informal care costs are excluded. The results for the TQ analysis were similar, with a flatter CEAC for QALYs (probability of cost-effectiveness at €50,000/QALY: 32%) and a higher probability of cost-effectiveness for DHI improvement at the same threshold (79%).

### Sampling uncertainty

**Fig. 4:**
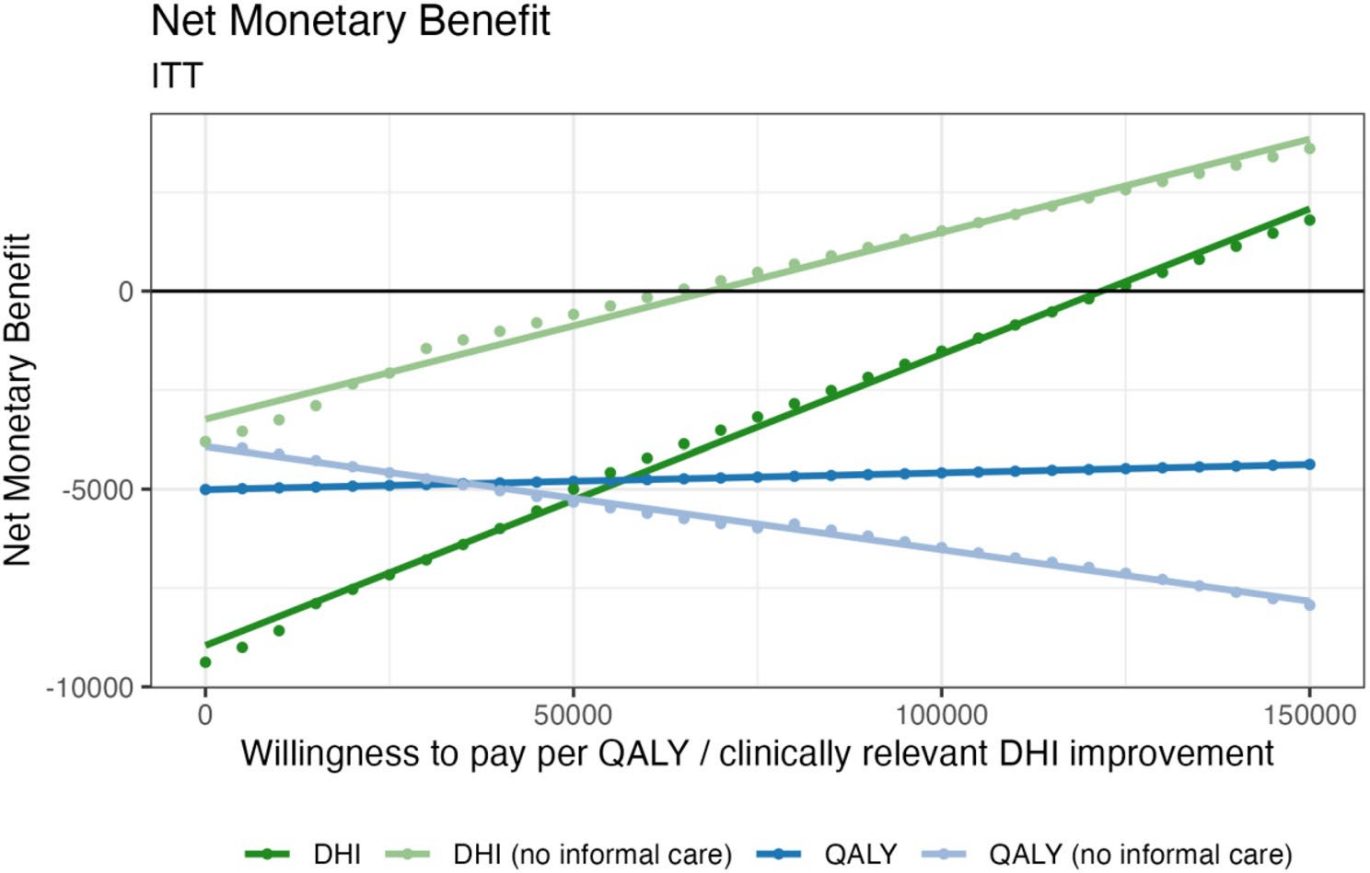
Net monetary benefit for the ITT; DHI: Dizziness Handicap Inventory, QALY: quality-adjusted life years

Sampling uncertainty, as measured by the NMB, yields similar results, as can be seen in Fig. 4. Uncertainty is considerable throughout; the 95% confidence intervals are wide and include zero across all WTP thresholds and all four estimation scenarios.

The NMB decreases when using QALY as the outcome denominator and informal care costs are included, reflecting the higher costs and lower QALYs of the IG. When including costs for informal care, the NMB increases only slightly with the willingness to pay and remains negative across all values of WTP for QALYs, reflecting the lack of cost-effectiveness of the intervention.

In contrast, the NMB curve for WTP per DHI improvement starts at €-9,379.76 (95% CI: -25,986.49;7,226.98) and remains negative up to a value of approximately €125,000 (estimate: 138.75 €/DHI improvement, 95% CI: -28,463.64;28,741.05), which corresponds to the ICER. Differences from the point estimate above are due to estimation uncertainty. Disregarding informal care cost shifts the curve upward, and the intervention is considered cost-effective starting from a WTP of approximately 65,000€/DHI improvement (estimate: 50.36 €/DHI improvement, 95% CI: -22,199.55;14,474.97).

## Discussion

The cost-effectiveness analysis conducted indicates that the intervention is dominated by optimised routine care in the ITT analysis from a societal perspective, as it is associated with higher costs and lower QALYs. However, when effectiveness is measured using disease-specific outcomes, the intervention demonstrates a favourable profile, with an ICER of 81,246.85€ per additional patient achieving a clinically relevant improvement in DHI scores. From a statutory payer perspective, costs were lower in the intervention group, although this was accompanied by a reduction in QALYs. Taken together, these findings suggest that the intervention improves disease-specific functioning without translating into measurable gains in generic health-related quality of life.

The results of this health economic evaluation are consistent with a RCT in a British primary care setting reporting small, if positive, QALY differences, which may point to the limited ability of VDB interventions to influence response behaviour to the EQ-5D. This underscores the value of disease-specific outcomes that are more responsive to changes in dizziness symptoms [41]. Similarly, a Dutch randomised trial reported minor QALY differences, but significant disease-specific outcomes, as well as ICERs and ICURs comparable to those observed in this study [42]. While the ICUR enables comparability across different healthcare interventions, the ICER provides a disease-specific measure that may be more relevant from a patient perspective.

A key driver of the observed cost differences was informal care, which accounted for the majority of total costs and remained higher in the intervention group throughout the study period. Informal care has been highlighted by prior research as a major contributor to total costs, particularly when functional independence limitations are under study [43, 44]. As informal care is closely linked to functional status, this result suggests that short-term improvements in dizziness symptoms may not immediately translate into reduced care needs, or may shift the burden from professional to informal caregivers.

Several factors may further explain the limited QALY gains observed. First, adherence to the intervention was low, with only seven patients following the CPW as intended. As fidelity was not formally assessed, the extent to which patients were exposed to the intervention remains unclear, and the relationship between adherence and outcomes cannot be evaluated. The present analysis therefore reflects the cost-effectiveness of offering the intervention to an eligible population rather than receiving it as intended. With more precise adherence data, future studies with more robust adherence monitoring could allow for a more precise evaluation of its causal effect. Second, the six-month follow-up period may have been insufficient to capture changes in health-related quality of life, particularly in patients with chronic vestibular disorders, where improvements may occur gradually over longer time horizons [45]. Third, the control group received an online training session for blinding purposes, which may have raised awareness of VDB management among participating GPs. This could have improved the quality of care delivered in the control group and thereby reduced the observable difference between groups, potentially leading to an underestimation of the intervention effect relative to routine care.

### Strengths and limitations

The ITT analysis relied more heavily on MICE to address missing data, whereas the TQ analysis required imputation for only 3.6% of values, representing a 71% reduction. While both approaches rely on different assumptions regarding the missingness mechanism, this consistency between findings from the ITT and TQ findings lends credibility to the imputation strategy employed in the primary analysis.

The intended recruitment target of 120 patients was not fully achieved, despite adjustments informed by prior feasibility studies [16]. Participants also had a relatively high baseline symptom burden with a mean DHI score of 53, indicating moderate-to-severe vestibular dysfunction, which may be associated with greater potential for improvement but also higher baseline care needs. The high proportion of female participants (71%) may further limit transferability to more gender-balanced populations.

Informal care was the primary cost driver and is particularly sensitive to self-report bias and individual differences in valuation [46]. The recall periods used in the FIMA questionnaire may not have aligned well with the study’s follow-up intervals, complicating precise cost estimation. By presenting analyses both with and without informal care costs, this study underscores the importance of transparency in economic evaluation. Notably, a recent systematic review [11] did not include informal care costs; however, the present findings suggest this omission may substantially underrepresent the true burden on caregivers of individuals with VDB.

Medication costs were not assessed, and evidence suggests pharmacological treatment plays a secondary role in VDB management, with one systematic review reporting an average of 1.8 drugs per patient and 61% of patients on medication [11]. Given that physiotherapy was the primary intervention target and DHI improvements suggest reduced symptom burden, medication costs may have been lower in the intervention group; if not, their exclusion likely introduces minimal bias.

Within the German healthcare context, the absence of a formal WTP threshold may allow for a more flexible interpretation of these findings. Unlike the United Kingdom, where the National Institute for Health and Care Excellence applies a threshold of £20,000-£30,000 per QALY [23], German decision-making can incorporate disease-specific outcomes and patient-relevant benefits [47]. In this context, the observed improvements in DHI scores may carry clinical relevance, though further research is needed to establish appropriate WTP thresholds for vestibular outcomes.

Finally, differences in reporting behaviour may have introduced cost variation unrelated to the intervention, and residual reporting biases cannot be ruled out despite adjustment for baseline costs, particularly in the context of cluster randomisation. An analysis of data from the Medical Expenditures Panel Survey reported annual per-patient cost of $2,658.73 (95% CI: 1,868.79;3,385.66) after controlling for sociodemographic characteristics [10], which is approximately half the total cost estimated through standardised unit costs in MobilE-PHY2. Future studies could benefit from adopting episode-based documentation as proposed by Laux et al. [48], to provide a more comprehensive understanding of patient care pathways.

## Conclusion

The CPW improved DHI scores, demonstrating its ability to reduce the impact of VDB on mobility, but these benefits were achieved at higher costs compared with optimised routine care. These improvements in disease-specific functioning did not translate into measurable gains in overall health-related quality of life or justify the intervention’s higher cost from a broader economic perspective. These findings are limited by our relatively small sample size, low adherence and the methodological challenges of capturing informal care costs in particular. Future research should prioritise the development and application of more sensitive, condition-specific tools to comprehensively assess the value of interventions, objective cost data from statutory health insurance records, and the development of appropriate WTP benchmarks for vestibular outcomes.

## Supplementary Information

Below is the link to the electronic supplementary material.

Supplementary Material 1

Supplementary Material 2

Supplementary Material 3

## Data Availability

All data generated or analysed and the measurements used during this study are available from the authors on request.

## Abbreviations

BI: Barthel-Index
TQ: Complete Cases
CEACs: Cost-effectiveness acceptability curves
CE-planes: Cost-effectiveness planes
CG: Control group
CHEERS: Consolidated Health Economic Evaluation Reporting Standards
CPW: Care pathway
cRCT: Cluster-randomised controlled trial
DHI: Dizziness Handicap Inventory
EQ-5D-5L: European Quality of Life Five-Dimension Five-Level scale
FIMA: Questionnaire for Health-Related Resource Use in an Elderly Population (Fragebogen zur Inanspruchnahme medizinischer und nicht-medizinischer Versorgungsleistungen im Alter)
GLMM: Generalized Linear Mixed Model
GP: General practitioner
ICER: Incremental cost-effectiveness ratio
ICUR: Incremental cost-utility ratio
IG: Intervention group
ITT: Intention-to-treat
MAR: Missing at random
MICE: Multiple Imputations by Chained Equations
MiniBESTest: Mini-Balance Evaluation Systems Test
MobilE-PHY2: Interdisciplinary care pathway for older patients with dizziness and balance disorders in primary
NMB: Net Monetary Benefit
QALYs: Quality-adjusted life years
PT: Physiotherapists
VDB: Vertigo, dizziness and balance disorders
WTP: Willingness-to-pay
