# Supplementary material for: Health economic evaluation of an interdisciplinary care pathway for older patients with vertigo, dizziness and balance disorders in primary care (MobilE-PHY2) - a cluster-randomised trial
Source: Cost Eff Resour Alloc. 2026 Jun 4;24:74. doi: 10.1186/s12962-026-00779-0 (PMC13244986; doi:10.1186/s12962-026-00779-0)
Supplement: Supplementary file 1 — Supplementary Material 1 [file 12962_2026_779_MOESM1_ESM.docx]

# **Appendix 1: TIDieR Checklist**

Health Economic Evaluation of an interdisciplinary care pathway for older patients with vertigo, dizziness and balance disorders in primary care (MobilE-PHY2) - a cluster-randomised trial

**1. Brief Name**:

Interdisciplinary care pathway for older patients with vertigo, dizziness and balance disorders in primary care (MobilE-PHY2)

**2.** **Why**:

Dizziness is among the most frequent reasons for consulting a general practitioner (GP), with prevalence estimates of GP consultations due to dizziness reaching up to 15.5% [1,2]. Despite its high prevalence, establishing a definitive diagnosis remains a significant challenge in the management of dizziness and vertigo, emphasising the need for effective, evidence-based interventions [3]. To address this gap, a multidisciplinary care pathway (CPW) was developed and piloted, integrating evidence-based approaches for dizziness and vertigo management. The pilot study demonstrated potential benefits for patients and highlighted satisfaction among GPs and physiotherapists (PTs) with the educational training provided. Patients also responded well to physiotherapy, with good treatment adherence reported. These findings suggest that a structured, multidisciplinary approach could enhance the management of dizziness and vertigo in primary care settings [4].

A logic model was developed for the intervention (Horstmannshoff et al., 2023), outlining key components for both professionals and patients. The intervention aimed to equip GPs and PTs with the necessary knowledge and skills to support patients in managing their symptoms more effectively. By improving patient self-management, the intervention was expected to reduce the need for healthcare resources, ultimately leading to lower healthcare costs.

**3.** + **4.** **What**:

GPs conducted anamnesis and examinations, referring patients when necessary, based on an evidence-based checklist. The initial assessment guided GPs through a structured evaluation, including symptom quality, frequency, and duration, as well as positional manoeuvres and neurological tests. The checklist provided recommendations for specialist referrals, which could include neurologists, otorhinolaryngologists, internists, cardiologists, or PTs. Treatment options encompassed symptomatic bridging therapy, medication, or general therapeutic adjustments such as medication optimization. Follow-up assessments were conducted at four weeks and three months. If a patient was referred to a participating PT, they provided physiotherapeutic care based on an evidence-based decision tree. The decision tree primarily focussed on vestibular rehabilitation but also included educational material, such as structured exercise programs and informational leaflets. The PT evaluated and documented their therapy of choice.

GP and PT received step-by-step instructions on the study process.

**5. Who Provided**:

GP are licensed physicians contracted by the association of statutory health insurance physicians (*Kassenärztliche Vereinigung*). Those in the intervention group received a two-hour intervention-specific online training.

Physical therapists completed at least three years of training and had to be state-licensed. PTs participated in a one-day intervention-specific training program.

**6. How**:

The intervention was provided individually and in person. Each patient received a one-on-one consultation with their GP. Appointments between PTs and patients are also one-on-one and face-to-face. Each patient had at least two consultations with a GP.

**7. Where**:

The intervention was conducted in two German study sites who have an urban centre with a rural environment (Dresden and Rosenheim).

**8. When and How Much**:

First, the patients had an inclusion interview with the GP. During the second visit, the checklist was applied by the GP, as well as during follow-up visits after 4 weeks and 3 months. If they were referred to a PT, the PT-based part of the intervention was applied upon their first appointment.

Eligible patients were at least 60 years old, exhibited VDB-related symptoms, were able to stand unaided, and able to give written informed consent. Additional inclusion criteria required a Dizziness Handicap Inventory (DHI) score of 12 or more, sufficient cognitive ability (Mini-Mental State Examination Score >= 20), and sufficient proficiency in German to complete study-related questionnaires. Patients were excluded if they had a life expectancy of less than one year, if their VDB symptoms were due to substance abuse, or if they had any of the following psychiatric disorders: F10.-F19, F20.-F29, F30., F31., F32.2, F32.3, F32.8, F32.9, F33.2, F33.3, F33.8, F33.9.

**9. Tailoring**:

No modifications or adaptations to the intervention were planned or implemented.

**10. Modifications**:

The intervention was not modified throughout the study duration.

**11. How Well**:
Intervention adherence and fidelity were not assessed.

**12. Actual**:

7 patients followed the CPW as intended.

## References

1. Bösner S, Schwarm S, Grevenrath P, Schmidt L, Hörner K, Beidatsch D, et al. Prevalence, aetiologies and prognosis of the symptom dizziness in primary care – a systematic review. BMC Fam Pract. 2018;19:33.

2. Wun Y, Lu X, Liang W, Dickinson J. The work by the developing primary care team in China: a survey in two cities. Fam Pract. 2000;17:10–5.

3. Stephan A-J, Kovacs E, Phillips A, Schelling J, Ulrich SM, Grill E. Barriers and facilitators for the management of vertigo: a qualitative study with primary care providers. Implement Sci. 2018;13:25.

4. Horstmannshoff C, Skudlik S, Petermann J, Kiesel T, Döringer T, Crispin A, et al. Effectiveness of an evidence-based care pathway to improve mobility and participation in older patients with vertigo and balance disorders in primary care (MobilE-PHY2): study protocol for a multicentre cluster-randomised controlled trial. Trials. 2023;24:91.
