# Supplementary material for: Health economic evaluation of an interdisciplinary care pathway for older patients with vertigo, dizziness and balance disorders in primary care (MobilE-PHY2) - a cluster-randomised trial
Source: Cost Eff Resour Alloc. 2026 Jun 4;24:74. doi: 10.1186/s12962-026-00779-0 (PMC13244986; doi:10.1186/s12962-026-00779-0)
Supplement: Supplementary file 2 — Supplementary Material 2 [file 12962_2026_779_MOESM2_ESM.docx]

# **Appendix 2: Description of implementation strategies**

Health Economic Evaluation of an interdisciplinary care pathway for older patients with vertigo, dizziness and balance disorders in primary care (MobilE-PHY2) - a cluster-randomised trial

Several implementation strategies were employed, they are described based on Proctor et al [1].

| **Name** | **Definition** | **Specification** |
| --- | --- | --- |
| GP educational training (intervention group) | Two-hour educational training (online) | A VDB expert and neuro-otologist delivered a two-hour educational training for GPs, and if requested their medical assistants, containing information on recent diagnostic and therapeutic developments for VDB in older patients, and an introduction into the use of the checklist. |
| GP educational training (control group) | 30 minutes educational training (online) | 30 minutes educational training containing information on the German national guideline "acute dizziness" [2]. |
| PT education training | One day training session (presence, hybrid) | A VDB expert and physiotherapist delivered a one-day hands-on training session for PTs, which included information on VDB, and the vestibular rehabilitation of older patients. The training enabled PTs to understand the decision tree and apply it. Afterwards, PTs were able to send treatment videos and receive feedback from the VDB expert conducting the training. |
| Telephone hotline | telephone support | The study teams in Dresden and Rosenheim provided the opportunity for GPs and PTs to answer questions on content and methodology of the study, and other problems or needs. |

## References

1. Proctor EK, Powell BJ, McMillen JC. Implementation strategies: recommendations for specifying and reporting. Implement Sci. 2013;8:139.

2. Abholz H-H, Jendyk R. Akuter Schwindel in der Hausarztpraxis. S3-Leitlinie. [Acute dizziness in general practice. S3 (evidence and consensus-based) guideline] [Internet]. Deutsche Gesellschaft für Allgemeinmedizin und Familienmedizin (DEGAM); 2018. Available from: https://www.degam.de/files/Inhalte/Leitlinien-Inhalte/Dokumente/DEGAM-S3-Leitlinien/053-018_Akuter%20Schwindel%20in%20der%20Hausarztpraxis/oeffentlich/053-018L_Akuter%20Schwindel%20in%20der%20Hausarztpraxis_redakt%20ueberarbeitet_20-4-2018.pdf
