# Supplementary material for: Health economic evaluation of an interdisciplinary care pathway for older patients with vertigo, dizziness and balance disorders in primary care (MobilE-PHY2) - a cluster-randomised trial
Source: Cost Eff Resour Alloc. 2026 Jun 4;24:74. doi: 10.1186/s12962-026-00779-0 (PMC13244986; doi:10.1186/s12962-026-00779-0)
Supplement: Supplementary file 3 — Supplementary Material 3 [file 12962_2026_779_MOESM3_ESM.docx]

# **Appendix 3: Calculation of costs and QALY**

Health Economic Evaluation of an interdisciplinary care pathway for older patients with vertigo, dizziness and balance disorders in primary care (MobilE-PHY2) - a cluster-randomised trial

### Calculation of costs

The number of days between the two questionnaires each participant completed was defined as the time under intervention. Unadjusted total cost at follow-up $C_{i}$ were calculated as individual total cost $C_{t_{2}}$times the proportion of a year spent under intervention:

$$C_{i}=C_{t_{2}}\cdot\frac{\text{days}\left( t_{0};t_{2} \right)}{365\text{days}}$$

To adjust for potential differences in baseline cost, the costs 12 months prior to intervention were calculated based on the $t_{0}$FIMA questionnaire.

### Calculation of QALYs

QALYs were weighted by the duration of the time under intervention, using linear interpolation between baseline and follow-up (i.e. the area-under-the-curve method).

$$\text{QALY}_{i}=\left[ \frac{\text{index}_{t_{0}}+\text{index}_{t_{2}}}{2}\cdot\text{days}\left( t_{0};t_{2} \right) \right]/365$$

Where $\text{index}_{t_{0}}$ and $\text{index}_{t_{2}}$ are the individual EQ-5D valuation indices measured at $t_{0}$ and $t_{2}$.
